# Clinical utility of inflammatory biomarkers in COVID‐19‐related sudden sensorineural hearing loss

**DOI:** 10.1002/iid3.1055

**Published:** 2023-10-18

**Authors:** Xu Zhang, Li Chen, Bing Guan

**Affiliations:** ^1^ Dalian Medical University Dalian China; ^2^ Department of Otolaryngology‐Head and Neck Surgery The Second People's Hospital of Yibin City Yibin China; ^3^ Department of Otolaryngology‐Head and Neck Surgery, Clinical Medical College Yangzhou University Yangzhou China

**Keywords:** COVID‐19, inflammatory markers, prognosis, risk factor, SARS‐CoV‐2, sudden sensorineural hearing loss

## Abstract

**Background:**

The etiology and pathophysiological mechanisms of sudden sensorineural hearing loss (SSNHL) remain unclear, but it is generally believed to be associated with viral infections, vascular diseases, and autoimmune disorders. Considering that coronavirus disease 2019 (COVID‐19) is promising candidates for SSNHL, we studied the immune cells changes by COVID‐19 in patients with SSNHL.

**Methods:**

We collected data from 47 patients with SSNHL and severe acute respiratory syndrome coronavirus 2 (SARS‐CoV‐2) positive. Patients were divided into ineffective or effective groups based on the degree of hearing recovery at discharge. Clinical information was collected and processed for both groups. Logistic regression models were used to determine the risk factors for an unfavorable prognosis in COVID‐19‐related SSNHL. Receiver operating characteristic (ROC) curves were used to estimate the predictive value.

**Results:**

There was statistically significant difference in C‐reactive protein (CRP), auditory curve, degree decline, pretreatment hearing, posttreatment hearing, systolic blood pressure, diastolic blood pressure, total bilirubin, neutrophil‐to‐lymphocyte ratio (NLR), systemic immune‐inflammation index (SII), indirect‐bilirubin and platelet count between groups (*p* < 0.05). In the logistic regression model, high levels of SII and NLR were associated with treatment ineffectiveness, pre‐ and postcorrectively (both, *p* < 0.05). And ROC curve analysis showed higher AUC of 0.765 for SII, 0.697 for NLR,0.681 for CRP, and 0.553 for platelet‐to‐lymphocyte ratio (PLR) in predicting treatment outcomes.

**Conclusion:**

The prognosis of COVID‐19‐related SSNHL was associated with inflammation. SII, NLR and CRP could serve as predictive markers of unfavorable outcomes in COVID‐19‐related SSNHL. SII may be considered an independent risk factor for poor prognosis in COVID‐19‐related SSNHL.

## Graphical abstract

The prognosis of COVID‐19‐related sudden sensorineural hearing loss (SSNHL) is associated with inflammation. λSystemic immune‐inflammation index (SII), neutrophil‐to‐lymphocyte ratio, and C‐reactive protein could serve as predictive markers of unfavorable outcomes in COVID‐19‐related SSNHL. λSII may be considered an independent risk factor for poor prognosis in COVID‐19‐related SSNHL.

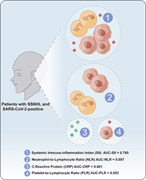

## 1 INTRODUCTION

Sudden sensorineural hearing loss (SSNHL), or sudden deafness (SD), is a sudden, unexplained sensorineural hearing loss that occurs within 72 h, with a decline of at least 30 decibels in three consecutive frequencies.^1^ Although the etiology and pathophysiological mechanisms of SSNHL are yet unknown, it is generally accepted that viral infections, vascular illnesses, and autoimmune disorders are connected to the condition.^2^ Rapid‐onset unilateral hearing loss, normal results on standard ear exams, and accompanying symptoms such ear congestion, tinnitus, and vertigo are typical clinical hallmarks of SSNHL. The most common main therapy is systemic corticosteroids.^2^, ^3^ With a sharp rise in incidence with increasing age,^4^ the estimated incidence of SSNHL in the United States is 27 cases per 100,000 people. Recent research has raised the possibility of a connection between SSNHL^5^, ^6^, ^7^ and coronavirus disease 2019 (COVID‐19) infection. The incidence of SSNHL was reported to be 23 cases per 100,000 people during the COVID‐19 pandemic.^8^ As the percentage of patients with diagnoses rose over that time, it is possible that fewer outpatient visits during the pandemic contributed to the decline in the total number of cases.^9^ During the COVID‐19 pandemic, SSNHL incidence rates were higher than they had been previously, suggesting that virus‐related variables may have contributed to the development of SSNHL.^10^

Central and peripheral auditory system abnormalities may be linked to hearing loss and auditory impairment in COVID‐19 individuals.^11^ Severe Acute Respiratory Syndrome (SARS) is a highly contagious and potentially life‐threatening respiratory illness caused by the SARS coronavirus (SARS‐CoV). SARS‐CoV‐2, commonly referred to as the coronavirus 2, is a big virus that has caused a global epidemic. Presently, the pathophysiological basis of SSNHL in individuals with COVID‐19 infection appears to be associated with four plausible mechanisms: (1) Cochlitis or neuritis arises due to viral infiltration of the inner ear or involvement of the vestibulocochlear nerve. (2) The stress reaction occurs as a result of a cross‐reactivity between an inner ear antigen and a viral infection. (3) COVID‐19 individuals exhibit a range of cardiovascular symptoms, including anomalous blood clotting. Due to the absence of collateral capillaries inside the labyrinthine artery in the cochlear microcirculation, cochlear hair cells exhibit a heightened vulnerability to ischemia injury. Consequently, individuals with this characteristic are at an increased risk of developing inner ear thrombosis or hypoxia. (4) Immune‐mediated diseases, characterized by excessive production of pro‐inflammatory cytokines.^12^ Therefore, inflammation is crucial in those with SSNHL caused by COVID‐19. The clinical traits and prognostic variables of SSNHL associated with COVID‐19 are not well studied.

Currently, there is only a small amount of research regarding the clinical characteristics and factors that influence treatment outcomes in cases with SSNHL that are complicated by COVID‐19. Following the initial outbreak in China, there has been a notable escalation in the quantity of reports pertaining to the outbreak. Additionally, certain individuals have exhibited a negative prognosis. Hence, we conducted a comparative analysis of the clinical characteristics and prognostic outcomes among individuals diagnosed with COVID‐19 and sudden deafness within a clinical cohort. Additionally, we examined pertinent inflammatory markers, thereby offering novel perspectives and potential avenues for the management and prognosis of sudden deafness in the context of viral infection.

## 2 METHODS

### 2.1 Data sources

From November 2022 to March 2023, 47 patients with COVID‐19 complicated with sudden deafness in the Clinical Medical College of Yangzhou University were retrospectively analyzed. The following were the criteria for inclusion: (1) Positive results for SARS‐CoV‐2 in polymerase chain reaction (PCR) testing of oropharyngeal and nasopharyngeal swabs; (2) Aged 18 years or older; (3) Sudden onset of sensorineural hearing loss within 72 h, with a minimum continuous decrease of ≥20 dBHL in at least three consecutive frequencies; (4) Mostly unilateral, with a few bilateral or sequential cases; (5) Patients may exhibit symptoms including tinnitus, ear stuffy, and echo; (6) Patients may experience disorientation, vertigo, nausea, and vomiting; (7) No prior treatment received before the onset of symptoms.

The exclusion criteria were as follows: (1) Patients with accompanying diseases or injuries that could cause hearing loss, such as Ménière's disease, chronic suppurative otitis media, other central nervous system diseases, head trauma, or the use of ototoxic drugs; (2) Patients were confirmed to have the posterior cochlear space‐occupying lesion as evidenced by magnetic resonance imaging (MRI) of the skull or middle inner ear, or computed tomography (CT) scan of the temporal bone; (3) Patients with a history of previous hearing loss or tinnitus; (4) Patients with a history of otologic pathology or ear surgery.

### 2.2 Treatment and therapeutic effect evaluation

In this investigation, all patients received comprehensive treatment, which included systemic corticosteroid therapy, intravenous administration of neurotrophic drugs, and dexamethasone was injected into the tympanic cavity of the affected ear, once a day, a total of three times, 5 mg each time. In accordance with the 2015 Chinese Medical Association's Guidelines for the Diagnosis and Treatment of Sudden Deafness,^13^ patients with “high‐frequency SSNHL (HF‐SSNHL)” and “total deafness of SSNHL (TD‐SSNHL)” received additional administration of the fibrinogen‐reducing drug, batroxobin, at an initial dose of 10 BU, followed by 5 BU. Before each administration, fibrinogen levels were measured to ensure they were above 1 g/L to prevent hemorrhage and other adverse effects. During the treatment regimen, the blood glucose levels of individuals diagnosed with diabetes were assessed on five separate occasions throughout the day. These measurements included postfasting, 2 h after each of the three main meals, and at 21:00 p.m. Blood pressure was assessed twice daily in hypertensive patients, with measurements taken both in the morning and in the evening. In instances where there are fluctuations in the patient's glycemia or blood pressure, prompt consultation and modification of the hypoglycemic or blood pressure treatment plan is undertaken by the endocrinologist or cardiologist. Furthermore, patients who experienced challenges in glycemic or blood pressure control had their systemic steroid therapy promptly ceased. It is imperative to underscore that the course of treatment is defined in this context as the duration of systemic steroids.

Before and after treatment, pure tone audiometry was conducted to evaluate the therapeutic effect. If the pure‐tone audiometric (PTA) of the affected frequencies improved by at least 15 dB or if the affected ear reached the same level as the normal or unaffected ear, the treatment was deemed efficacious. If the improvement in PTA was less than 15 dB, the treatment was deemed ineffective.

### 2.3 Clinical information collection

Patients' baseline characteristics (age, gender, height, weight, history of diabetes, and history of hypertension), vital signs (systolic blood pressure [SBP], diastolic blood pressure [DBP]), clinical history, SSNHL‐related information, and laboratory parameters were retrieved from hospital records. Body mass index (BMI) was calculated using the following formula: BMI = Weight (Kilograms)/Height (Meters)^2^ Each case was assigned a neutrophil‐to‐lymphocyte ratio (NLR), platelet‐to‐lymphocyte ratio (PLR), and systemic immune‐inflammation index (SII). SII was calculated using the following formula: SII = N × P/L. The research was sanctioned by the Hospital Ethics Committee (2023ky052).

### 2.4 Statistical analysis

The chi‐square test was utilized to analyze count data presented as percentages (*n*, %). Rank‐sum tests were utilized to analyze ranked data. Continuous variables with a normal distribution were expressed as mean standard deviation (SD) and compared using *t*‐tests on samples from independent groups. Alternately, quartiles (M, Q25, and Q75) were used to represent continuous variables that did not have a normal distribution, and rank‐sum tests were used to compare between groups. On the basis of clinical indicators of patients, logistic regression models were used to control for potential confounding variables. Model I included adjustments for age, gender, and diabetes. Model II added SBP and DBP to Model I as additional modifications. Model III added TBiL, indirect bilirubin, Cr, and cholesterol to Model II as adjustments. To assess the predictive value of SII, NLR, PLR, and CRP for treatment outcomes, receiver operating characteristic (ROC) curves were constructed. The significance level was set to 0.05, and a test with two hypotheses was conducted. SPSS 26.0 was used to perform statistical analysis.

## 3 RESULTS

In this study, among the 47 eligible patients, 28 were classified as ineffective responders and 19 as effective responders, yielding an effective treatment rate of 40.43%. Comparing clinical data from patients with different treatment outcomes revealed no statistically significant differences in gender, hypertension, cardiovascular disease, smoking, drinking, etiology cause, ear stuffiness, tinnitus, swirl, dizziness, treatment time, PLR, total bilirubin (TBiL), albumin, lymphocyte count, creatinine (Cr), platelet volume, Fibrinogen (FIB), cholesterol, triglyceride, high‐density lipoprotein (HDL), low‐density lipoprotein (LDL), BMI, D‐Dimer (DD), and glycosylated hemoglobin, Type A1C (Hba1c) (*p* > 0.05). The ineffective group had a slightly higher average age (53.32 ± 12.25 vs. 46.05 ± 17.10) and a higher prevalence of diabetes (21.4% vs. 0.0%) compared to the effective group, but these differences were not statistically significant (*p* > 0.05). The ineffective group had significantly higher levels of CRP (14.64 ± 8.02 vs. 9.35 ± 6.34) and showed significant differences in the auditory curve (*p* < 0.05). The ineffective group had a higher proportion of patients with a degree decline of 2 (92.9% vs. 57.9%), which was statistically significant (*p* < 0.05). The ineffective group had higher pretreatment hearing (83.25 ± 14.49 vs. 68.42 ± 19.40) and posttreatment hearing (81.61 ± 14.34 vs. 35.58 ± 20.77) compared to the effective group, and these differences were statistically significant (*p* < 0.05). Additionally, compared to the effective group, the ineffective group had higher levels of SBP (136.61 ± 17.07 vs. 125.47 ± 8.39), DBP (85.79 ± 7.35 vs. 77.05 ± 5.45), TBiL (17.02 ± 5.04 vs. 6.21 ± 1.00), NLR (5.86 ± 2.89 vs. 4.08 ± 1.54), SII (1129.24 ± 693.55 vs. 595.75 ± 275.36), indirect bilirubin (12.58 ± 4.95 vs. 3.73 ± 0.70), and platelet count (198.14 ± 77.54 vs. 146.95 ± 40.05), with statistically significant differences (*p* < 0.05) (Table 1).

**Table 1:** Comparison of clinical data of patients with different therapeutic effects.

Project	Ineffective group (*n* = 28)	Effective group (*n* = 19)	*p*
Age (mean ± SD)	53.32 ± 12.25	46.05 ± 17.10	0.096
Gender = male (%)	12 (42.9)	10 (52.6)	0.718
Hypertension (%)	5 (17.9)	3 (15.8)	1.000
Diabetes (%)	6 (21.4)	0 (0.0)	0.086
Cardiovascular (%)	1 (3.6)	0 (0.0)	1.000
Day (mean ± SD)	7.64 ± 6.46	5.74 ± 4.38	0.268
Smoke (%)	2 (7.1)	0 (0.0)	0.650
Drink (%)	1 (3.6)	0 (0.0)	1.000
Auditory curve (LF‐SSNHL/HF‐SSNHL/FT‐SSNHL/TD‐SSNHL, %)	0/4/4/20	4/3/6/6	0.013
Degree decline = Severe to profound (%)	26 (92.9)	11 (57.9)	0.012
Pretreatment hearing (mean ± SD)	81.25 ± 14.49	68.42 ± 19.40	0.004
Pro‐treatment hearing (mean ± SD	81.61 ± 14.34	35.58 ± 20.77	<0.001
Cause (%)	1 (3.6)	3 (15.8)	0.347
Ear stuffy (%)	5 (17.9)	9 (47.4)	0.065
Tinnitus (%)	24 (85.7)	15 (78.9)	0.833
Swirl (%)	12 (42.9)	3 (15.8)	0.102
Dizziness (%)	11 (39.3)	4 (21.1)	0.319
Treatment time (mean ± SD)	7.29 ± 2.00	6.63 ± 1.30	0.216
SBP (mean ± SD)	136.61 ± 17.07	125.47 ± 8.39	0.012
DBP (mean ± SD)	85.79 ± 7.35	77.05 ± 5.45	<0.001
TBil (mean ± SD)	17.02 ± 5.04	6.21 ± 1.00	<0.001
CRP (mean ± SD)	14.64 ± 8.02	9.35 ± 6.34	0.020
NLR (mean ± SD)	5.86 ± 2.89	4.08 ± 1.54	0.018
PLR (mean ± SD)	164.87 ± 94.93	138.30 ± 64.09	0.293
SII (mean ± SD)	1129.24 ± 693.55	595.75 ± 275.36	0.003
Indirect bilirubin (mean ± SD)	12.58 ± 4.95	3.73 ± 0.70	<0.001
TP (mean ± SD)	66.67 ± 5.79	65.88 ± 3.38	0.593
Albumin (mean ± SD)	40.03 ± 6.90	41.69 ± 2.23	0.317
Lymphocyte (mean ± SD)	1.38 ± 0.62	1.21 ± 0.46	0.305
Neutrophil (mean ± SD)	6.76 ± 2.05	4.35 ± 0.91	<0.001
Cr (mean ± SD)	69.25 ± 32.45	63.95 ± 9.74	0.494
Platelet (mean ± SD)	198.14 ± 77.54	146.95 ± 40.05	0.011
Platelet volume (mean ± SD)	11.38 ± 1.71	11.64 ± 1.20	0.562
FIB (mean ± SD)	3.94 ± 5.58	3.26 ± 0.73	0.600
Cholesterol (mean ± SD)	4.60 ± 1.11	5.16 ± 0.86	0.069
Triglyceride (mean ± SD)	1.05 ± 0.62	1.22 ± 0.76	0.395
HDL (mean ± SD)	1.36 ± 0.35	1.37 ± 0.29	0.851
LDL (mean ± SD)	3.09 ± 1.03	3.21 ± 0.76	0.668
BMI (mean ± SD)	23.66 ± 2.14	23.12 ± 1.27	0.324
DD (mean ± SD)	0.44 ± 0.53	0.36 ± 0.35	0.594
HbA1c (mean ± SD)	5.90 ± 0.91	5.99 ± 0.70	0.708

Abbreviations: BMI, body mass index; Cr, creatinine; CRP, C‐reactive protein; DBP, diastolic blood pressure; DD, D‐dimer; FIB, fibrinogen; HbA1c, glycated hemoglobin; HDL, high‐density lipoprotein; LDL, low‐density lipoprotein; NLR, neutrophil to lymphocyte ratio; PLR, platelet to lymphocyte ratio; SBP, systolic blood pressure; SII, systemic inflammation index; TBiL, total bilirubin; TP, total protein.

In the unadjusted logistic regression models, high levels of SII (odds ratio [OR] = 1.003, 95% confidence intervals [CIs]: 1.001–1.005, *p* = 0.005) and NLR (OR = 1.454, 95% CIs: 1.083–1.952, *p* = 0.005) were associated with treatment ineffectiveness. After adjusting for other confounding factors, this study used three logistic regression models to explore the relationship between SII, NLR, and treatment outcomes (Table 2). In Model I, after adjusting for age, gender, and diabetes, high levels of SII (OR = 1.003, 95% CIs: 1.001–1.005) and NLR (OR = 1.454, 95% CIs: 1.083–1.952) remained significantly associated with treatment ineffectiveness (*p* = 0.005, 0.013). In Model II, SBP and DBP were added as additional adjustments to Model I, and it was found that high levels of SII (OR = 1.004, 95% CIs: 1.001–1.080) and NLR (OR = 1.392, 95% CIs: 1.055–1.837) were also significantly associated with treatment ineffectiveness (*p* = 0.009, 0.019). In Model III, TBiL, indirect bilirubin, Cr, and cholesterol were added as adjustments to Model II, and it was found that high levels of SII (OR = 1.013, 95% CIs: 1.001–1.006) and NLR (OR = 1.347, 95% CIs: 1.046–1.735) remained significantly associated with treatment ineffectiveness (*p* = 0.013, 0.021) (Table 2).

**Table 2:** SII levels and effect.

Variable	Unadjusted model		Model I		Model II		Model III	
	Odds ratio (OR) (95% confidence interval [CI])	*p*	OR (95% CIs)	*p*	OR (95% CIs)	*p*	OR (95% CIs)	*p*
Systemic immune‐inflammation index (SII)	1.003 (1.001–1.005)	0.005	1.003 (1.001–1.005)	0.005	1.004 (1.001–1.08)	0.009	1.003 (1.001–1.006)	0.013
Neutrophil‐to‐lymphocyte ratio	1.454 (1.083–1.952)	0.013	1.495 (1.089–2.052)	0.013	1.392 (1.055–1.837)	0.019	1.347 (1.046–1.735)	0.021

*Note*: Model I adjusted for age, gender, and diabetes; Model II adjusted for Model I added systolic blood pressure (SBP) and diastolic blood pressure (DBP); Model III adjusted for Model II added TBil, indirect bilirubin, Cr, and cholesterol.

**Figure 1:**
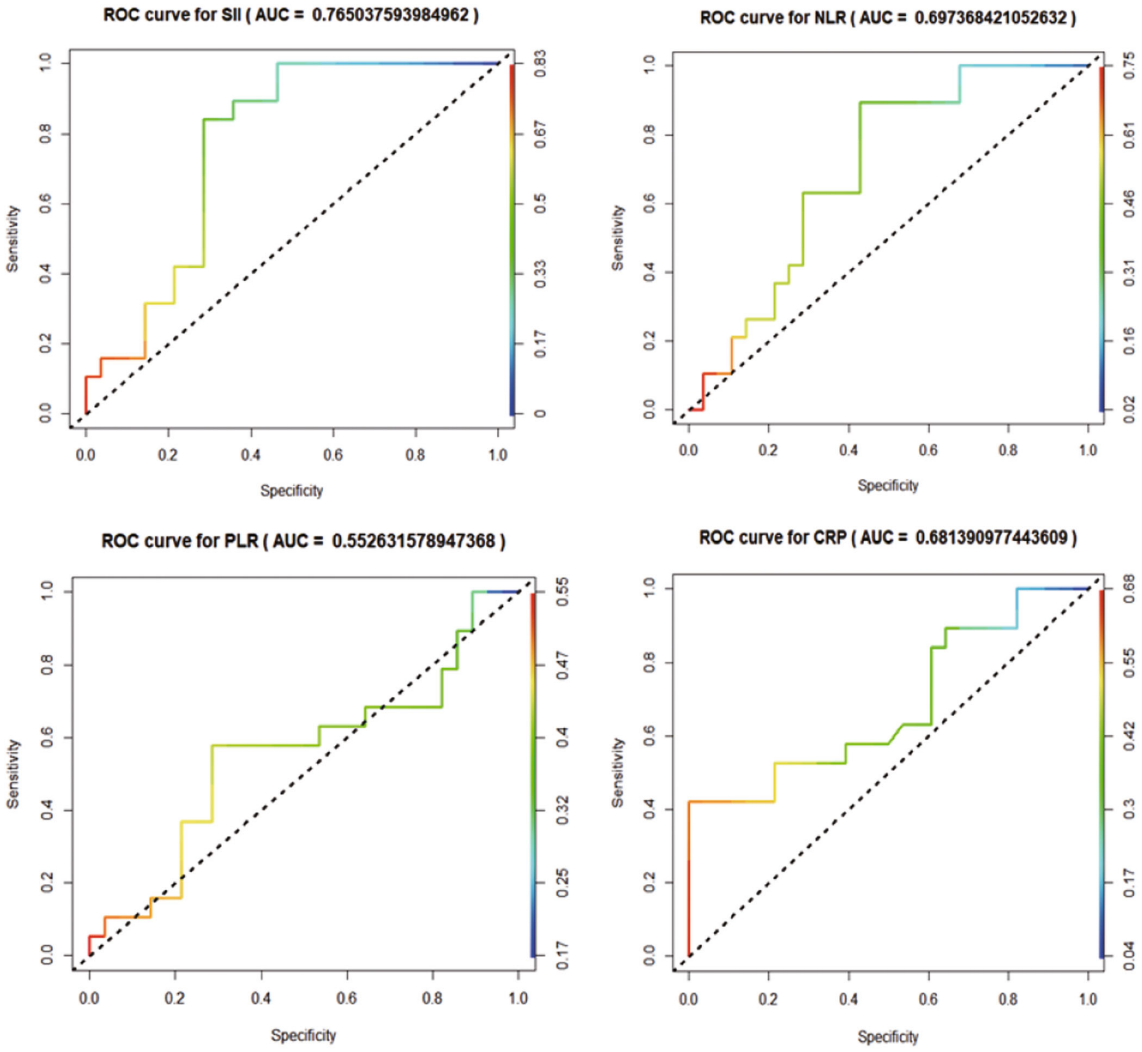
Receiver operating characteristic (ROC) curves of systemic immune‐inflammation index (SII), neutrophil‐to‐lymphocyte ratio (NLR), platelet‐to‐lymphocyte ratio (PLR), and C‐reactive protein (CRP) predicting treatment ineffectiveness.

ROC curves were established to evaluate the predictive value of SII, NLR, PLR, and CRP for treatment ineffectiveness. The results showed the area under the curves (AUC) of 0.765 for SII, 0.697 for NLR, 0.681 for CRP, and 0.553 for PLR, indicating that PLR had a lower predictive value, while NLR and CRP had some predictive value for treatment ineffectiveness, and SII had a good predictive value for treatment ineffectiveness (Figure 1).

## 4 DISCUSSION

This study involved a retrospective analysis of 47 patients who presented with acute deafness associated with COVID‐19. The study observed a range of median ages between 46 and 54. The sample consisted of 22 male participants and 25 female participants, with no statistically significant variation in incidence rates across genders. These findings align with prior studies.^14^, ^15^ A total of 15 individuals, including 32% of the sample, exhibited chronic ailments such as hypertension, diabetes, and cardiovascular disease, among others. These findings match with the results reported by Vural Fidan et al. in their study.^14^ Furthermore, it should be noted that one patient had a prior history of alcohol consumption, while two patients had a documented history of tobacco use. Additionally, four patients reported experiencing symptoms such as exhaustion, exposure to noise, and other relevant factors before the manifestation of the condition. The individuals in this study had different levels of hearing impairment, along with associated symptoms such as tinnitus (*n* = 39, 83.9%), vertigo (*n* = 15, 32.9%), dizziness (*n* = 15, 32.9%), and ear tightness (*n* = 14, 29.8%). The prevalence of tinnitus is shown to be high, as indicated by Xiangming Meng et al.^15^

To determine the factors influencing the prognosis of COVID‐19 patients with SSNHL, 47 patients were divided into effective or ineffective group. The results of these two groups demonstrated that the type of audiometric curve at the onset of SSNHL and the degree of hearing loss were related to the prognosis of COVID‐19 patients with SSNHL. The recovery rate for low‐frequency SSNHL was greater compared to flat‐frequency SSNHL and total impairment of SSNHL in patients with severe and profound hearing loss on their initial audiogram. Consistent with previous research on the prognostic factors of SSNHL, these findings indicate that patients with more severe hearing loss tend to have poorer recovery outcomes.^16^ In our study, the ineffective group had substantially higher levels of neutrophils, platelets, CRP, NLR, and SII than the effective group, indicating a greater inflammatory burden among patients with a poor prognosis. We found significant differences between the effective and ineffective groups regarding SII and NLR, which were independently associated with the prognosis of COVID‐19 patients with SSNHL after adjusting for other factors. Consequently, these two markers should be considered in the treatment of COVID‐19 patients with SSNHL to enhance management and prognosis.

PLR has been demonstrated to be predictive of cardiovascular diseases such as peripheral arterial occlusive disease.^17^ PLR levels were not associated with the prognosis of COVID‐19 patients with SSNHL in our study.

The NLR, or Neutrophil‐to‐Lymphocyte Ratio, is a recognized indicator that may be utilized to assess the presence of inflammation in both cardiac and noncardiac disorders.^18^ On the other hand, the SII, or Systemic Immune‐Inflammation Index, is a recently developed inflammatory marker that shows promise as a prognostic indicator in malignant tumors and inflammatory conditions.^19^ C‐reactive protein (CRP), a biomarker associated with acute phase inflammation, is frequently employed for the assessment of therapeutic effectiveness.^20^

NLR and CRP had values between 0.65 and 0.70 based on the area under the ROC curves, implying a moderate correlation with a poor prognosis in COVID‐19 patients with SSNHL. These two markers play a role in prognosis prediction. However, the area under the ROC curve for SII was greater than 0.70, indicating that it is a reliable indicator for predicting the prognosis of COVID‐19 patients with SSNHL. Patients with elevated NLR, CRP, and SII should therefore consider corticosteroid use a necessary intervention. Nevertheless, corticosteroids may have adverse effects. Therefore, for patients with extremely high SII values or other COVID‐19 complications, alternative treatment methods such as high‐dose intravenous methylprednisolone therapy (200 mg for 3 days) can be considered, or they can be referred to the appropriate departments for anti‐inflammatory treatment. We also recommend investigating additional inflammatory markers in patients with high SII suspected of viral infection to understand further the relationship between inflammatory markers and viral COVID‐19‐related SSNHL. In addition, COVID‐19 patients with elevated NLR, CRP, and SII levels should be vigilant for concurrent SSNHL, and appropriate measures, such as anti‐inflammatory treatment, should be implemented to facilitate early intervention and reduce disability rates.

### 4.1 Limitation

In this study, the peripheral blood inflammatory markers of COVID‐19 patients with SSNHL were evaluated. The assessment of hearing loss was conducted exclusively through the utilization of pure tone audiometry (PTA), in accordance with the 2015 Chinese Guidelines for the Diagnosis and Treatment of Sudden Deafness. This particular method of hearing assessment is widely employed and recognized as the most prevalent approach. In accordance with the guidelines outlined in the International Audiology report, it is important to take into account the speech recognition score (SRS) as a measure. The SRS not only provides information about the patient's hearing ability, but also offers insights into the functional status of their auditory nerve conduction pathway and auditory center.^21^ However, many medical centers do not use SRS as a routine inspection in China. The follow‐up period for these patients was brief, the sample size was small, and the patient population was restricted to a particular region. Furthermore, there is a lack of clarity regarding the correlation between the timing of COVID‐19 diagnosis and the development of SSNHL. It is worth noting that SSNHL might manifest before to the diagnosis of COVID‐19 or even during the recovery phase.^15^

To enhance the existing understanding of the clinical manifestations and inflammatory indicators linked to SSNHL in the context of COVID‐19, future investigations should encompass a more extensive cohort of COVID‐19 patients affected by SSNHL. Additionally, it would be valuable to gather data on patients with sudden‐onset sensorineural hearing loss (SRS) and their respective time of COVID‐19 diagnosis.

It will aid in early intervention and disability rate reduction, as well as facilitate individualized treatment.

## 5 CONCLUSION

Our research indicates that inflammation is linked to the prognosis of COVID‐19 patients with SSNHL. Patients with severe and pervasive hearing loss on the initial audiogram, those with low‐frequency SSNHL, and those with elevated levels of NLR, CRP, and SII may have a poor prognosis, with CRP, NLR, and SII being predictive of poor outcomes. SII is an independent risk factor associated with a poor prognosis in COVID‐19 SSNHL patients. Consequently, it is our recommendation for giving high‐dose steroid therapy to individuals exhibiting elevated levels of inflammatory markers. The risks and benefits of corticosteroid treatment must be evaluated for patients with elevated NLR, CRP, and SII levels, and alternative treatments or anti‐inflammatory therapies may be utilized. In addition, patients with COVID‐19 should closely monitor these inflammatory markers. Patients with elevated levels of NLR, CRP, and SII were subjected to early anti‐inflammatory intervention and individualized treatment to reduce the risk of SSNHL and its impact on disability. Additional large‐scale studies are required to better comprehend the clinical characteristics of SSNHL caused by viral infection, as well as the significance and predictive value of SII and other inflammatory markers.

## AUTHOR CONTRIBUTIONS

**Xu Zhang**: Conceptualization; data curation; formal analysis; investigation; methodology; resources; writing—original draft. **Li Chen**: Data curation; formal analysis. **Bing Guan**: Funding acquisition; supervision; validation; writing—review and editing.

## CONFLICT OF INTEREST STATEMENT

The authors declare no conflict of interest.

## ETHICS STATEMENT

The study was approved by the Ethics Committee of the Ethics Review Committee of the Clinical Medical College, Yangzhou University (Approval number: 2023ky052) and conducted according to the Declaration of Helsinki.

## Data Availability

The datasets generated and analyzed during the current investigation are available upon reasonable request from the corresponding author.
